# Absorption, Distribution and Excretion of ^14^C-Probimane in Mice Bearing Lewis Lung Carcinoma

**DOI:** 10.3797/scipharm.1005-05

**Published:** 2010-06-15

**Authors:** Da-Yong Lu, Rui-Ting Chen, Ting-Ren Lu, Hong-Ying Wu, Rong-Xin Qu, Jin-Yu Che, Bin Xu

**Affiliations:** 1 Shanghai University, Shanghai 200444, PR China; 2 Shanghai Institute of Materia Medica, Chinese Academy of Sciences, Shanghai 201203, PR China; 3 College of Science, Shanghai University, Shanghai 200444, PR China

**Keywords:** Anti-cancer agents, Bisdioxopiperazine compounds, Cancer chemotherapy, Probimane, Drug distribution

## Abstract

Spontaneous neoplasm metastasis, a fatalist pathological feature of cancer, is a long-evolving, multi-steps process that can now only be treated or controlled by drugs or immuno-modulators. Probimane (Pro), as a representative of the well-known class of antimetastatic agents ‘Bisdioxopiperazine compounds (Biz)’, is systematically studied for its absorption, distribution and excretion in mice bearing Lewis lung carcinoma by a radioactivity-detective method in this investigation. It is found that the ^14^C-Pro concentrations in different normal organs of mice at 2 hrs are very high and dramatically declined at 24 and 48 hrs. However, Pro concentrations in metastatic foci are slightly changed at the same time. Almost no change of Pro concentrations is observed in pulmonary metastatic nodules within 48 hrs. This evidence can be used to explain the characteristics of good metastatic inhibition by Biz compounds. The radioactivity in brain is relatively low because Pro can hardly penetrate into the blood-brain-barrier to eliminate brain tumors. The excretion of ^14^C-Pro is observed at the same ratios from both urine and feces and also at constant rates. These data are much useful for better understanding of the general pharmacological characters and possible antimetastatic mechanisms of actions of probimane and other Biz compounds from a new perspective and research angles.

## Introduction

There have been two most difficult problems in cancer biology and therapeutics, neoplasm metastasis and multi-drug-resistances (MDR). Among these two thorny problems, treatments of neoplasm metastasis are especially difficult and should be placed on higher agenda of the highest for its fatalist pathogenesis features and unpredictability of therapeutic outcome at the stage of drug initiation. Also, metastasized tumors often concomitantly manifest the characters of MDR. The treatment investigation of neoplasm metastases is one of the important topics for therapeutic researchers [1–3]. Bisdioxopiperazine compounds (Biz), including ICRF-154, Razoxane (ICRF-159, *Raz*), ICRF-186 and ICRF-187 (two stereo-isomers of Raz) and ICRF-193, developed in the UK, have been a series of serendipitous agents found to be effective against a model of spontaneous metastasis (Lewis lung carcinoma, 3LL) [4, 5]. Since Biz compounds are unique and conservative in pharmacological actions, their new analog Probimane [4,4′-(propane-1,2-diyl)bis[1-(morpholin-4-ylmethyl)piperazine-2,6-dione; AT-2153, Pro] [6–9] was synthesized at the Shanghai Institute of Materia Medica, Chinese Academy of Sciences, Shanghai, China. Since previous data showed that Pro has good antiproliferative effects *in vitro* [8] and moderate anticancer activity against the growth Lewis lung carcinoma *in vivo* [7], further work to specialized determinations of these characters is imperative and helpful to us.

The researches of anti-metastatic drugs, though at the odds nowadays facing, will be the hottest spot and discipline in the future. This work should carefully study the fate of ^14^C-probimane to help our understanding and knowledge about metastasis treatments of Biz compounds from drug distributions between normal organs, tumor tissues and metastatic foci.

## Materials and methods

**Fig. 1.**
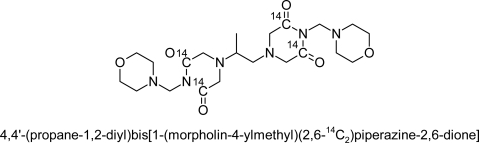
The structural formulae of ^14^C-Probimane

Drugs and Reagents: ^14^C-Pro (Specific activity 130kBq.mg^−1^) (Figure 1) were prepared by Prof. Xin Zhang at the Department of Medicinal Chemistry, Shanghai Institute of Materia Medica, Chinese Academy of Science and suspended in normal saline before use.

Animals and Tumor Models: C57BL/6j strain mice were purchased from Shanghai Center of Laboratory Animal Breeding, Chinese Academy of Sciences and experimentations were conducted in compliance with the Guidelines for the Care and Use of Research Animals, NIH, established by the Washington University’s Animal Studies Committee.

C57BL/6j mice (mean body weight 21.6g) were implanted sc with LLC (5×10^6^ cells) from donor mice. ^14^C-Pro 12mg was dissolved in 1 ml of normal saline at normal temperature. On day 17, mice were orally administered with ^14^C-Pro 120mg/Kg (about 0.2ml drug per mouse) and all mice were unlimitedly given food and water during the whole experiment. All mice were collected for their urine and feces in specialized-tailored glassy cages containing bottom tube for urine collection and intermediate mesh for collecting feces and these mice were sacrificed 2, 24 and 48 hrs after the injection of ^14^C-Pro. Then the blood (20μl), different organs of mice, primary tumors and pulmonary metastatic foci (approximately 3–10mg each – carefully weight and record organ weight) were separately collected in radioactive isotope couture. Exactly 0.1ml of formic acid and H_2_O_2_ were separately added to each testing-tube, then change in temperature up to 80°C for 45min for total digestion of tissues was observed. Then 5ml of ethylene glycol mono-ethyl/xylene mixture containing 0.5%PPO (5-phenyloxazolyl benzene) and 0.01%POPOP {1,4-bis-2-(5-phenyloxazolyl)benzene} was added and radioactivity in the counter was detected by an auto-liquid-scintillate (YSJ-78) PR China.

The radioactivity of urine and feces were also detected after 24 and 48hrs in the same way. This experiment was triplicated and all the data were statistically treated.

## Results

### The absorption and distribution of ^14^C-probimane in mice:

The distribution of ^14^C-Pro in mice bearing Lewis lung carcinoma (3LL) is manifested in Table1. Table1 showed that the extents of ^14^C-Pro in different normal organs of mice at 2 hrs were very high and dramatically declined at 24 and 48 hrs. Only about 1/10 of Pro remained after ^14^C-Pro was given at 48hrs comparing with those at 2hrs only except bowel and metastatic tumors. A persistent accumulation of Pro in metastatic tumors was observed. However the radioactivity in metastatic foci was slightly changed within 2 days. Almost no change in the Pro concentrations was observed in pulmonary metastatic nodules in the whole experiment and maintained in a high data within 48hrs. However, the radioactivity in primary tumors, though better than other normal tissues declined faster than metastatic tumors. The Pro ratios of metastatic tumor over primary tumor were 2.0, 4.4 and 15.8 at 2, 24 and 48 hrs, respectively. This evidence is a useful piece of information for the explanation of high inhibitions against neoplasm metastases by Pro and other Biz compounds. In addition, the ^14^C-Pro in brain was relatively low. It suggests that ^14^C-Pro might hardly penetrate into the blood-brain-barrier to eliminate and kill brain tumors or metastasized tumors unto brains. The ^14^C-Pro concentrations in gall bladders and intestines of mice at 2hrs are also very high. It suggests that ^14^C-Pro is soluble in both aquatic and organic tissues of the bodies. The ^14^C-Pro concentration in blood was declined very fast and only about 1% of ^14^C-Pro maintained in blood after 48hrs compared with those in 2hrs.

### The excretion of ^14^C-Pro:

The excretion of ^14^C-Pro was almost at the same ratios from urine and feces and in constant rates within the whole experiment Table2. It is a useful excretive data for us to understand the general characteristics and functions of Pro in living-bodies.

## Discussion

Since tumor metastasis is responsible for more than 60% of cancer deaths worldwide, metastasis therapy, as a result, remains to be one of the biggest challenges among all cancer therapeutics researches. Biz compounds are well-known for their antimetastasis action and several of their putative mechanisms [10–14]. However, their new modes of action and therapeutic profiles remain to be investigated for further understanding and proposition for their potential clinical availability. This work is a typical one. The inhibition of neoplasm metastasis by Raz has been previously explained as a result of reduction in the formation of blood vessels in primary tumor tissues (neovasculature) [10–12]. Thus, it has been proposed that Raz remains ineffective against the pulmonary metastases of 3LL (formed metastatic foci) [15] and nevertheless Pro can be more effective in the inhibition of neoplasm metastasis than Raz in formed metastatic foci through stronger antiproliferative effect [8, 9] and drug accumulation. From this data of ^14^C-Pro tracing, an obvious greater accumulation of Pro was found in tumor tissues, especially in metastatic foci. It can help to explain why Pro might effectively inhibit pulmonary metastases of Lewis lung carcinoma by its higher accumulation and affinity to metastatic nodules which lead further into higher cytotoxicity against formed metastatic foci in living-bodies.

This work shows that Pro can hardly penetrate into the blood-brain-barrier, and it suggests Pro might be less effective to brain tumors or against those other tumors which have been metastasized into the brains. This result is very similar with previously reported data of Raz by Greig N *et al* determined by a HPLC assay [16].

## Figures and Tables

**Tab. 1.** Radioactivity distributions (Probimane concentrations) in different tissues of mice bearing Lewis lung carcinoma by ig ^14^C-probimane 120mg/Kg

**Tissues**	**Probimane concentrations (ng/g tissues)**		
	**2 hrs**	**24 hrs**	**48 hrs**
Brain	17.8 ± 2.0	9.7 ± 3.0	2.2 ± 1.0
Skin	163.1 ± 10.9	67.2 ± 5.8	25.3 ± 1.7
Muscle	122.6 ± 15.4	34.0 ± 3.1	15.0 ± 1.5
Bone	90.1 ± 11.3	48.0 ± 7.3	15.1 ± 2.8
Gall bladder	648.7 ± 59.9	92.7 ± 10.0	26.9 ± 2.3
Primary tumor	70.1 ± 5.6	24.1 ± 2.6	7.7 ± 1.1
Metastatic tumor	142.3 ± 6.9	106.3 ± 7.0	121.5 ± 9.7
Heart	124.7 ± 13.6	22.8 ± 1.9	7.2 ± 0.7
Lung	149.5 ± 12.2	38.5 ± 2.9	29.2 ± 3.2
Testis	444.5 ± 25.0	46.5 ± 3.6	20.0 ± 2.5
Liver	370.6 ± 26.7	39.0 ± 4.7	15.3 ± 3.3
Spleen	365.3 ± 16.4	22.4 ± 2.3	15.9 ± 4.1
Kidney	446.8 ± 32.5	117.4 ± 9.9	22.7 ± 2.0
Stomach	1155.3 ± 45.7	107.2 ± 12.3	20.1 ± 2.5
Bowel	261.8 ± 17.4	60.1 ± 4.7	122.3 ± 14.6
Blood	202.4 ± 15.7	27.6 ± 2.0	3.3 ± 0.5

**Tab. 2.** The radioactivity excretion from mice bearing Lewis lung carcinoma by ig ^14^C-probimane 120mg/Kg

**Time (hrs)**	**Excretion (%)**		
	**Urine**	**Feces**	**Total**
24	18.7 ± 1.7	16.5 ± 1.5	35.2 ± 3.0
48	33.9 ± 2.5	32.0 ± 2.8	65.9 ± 4.4
